# Neuropathic Pain in Hand Osteoarthritis: A Cross-Sectional Study

**DOI:** 10.3390/jcm10194439

**Published:** 2021-09-27

**Authors:** Nico Magni, Jill Collier, Peter McNair, David A. Rice

**Affiliations:** 1 Department of Physiotherapy, School of Clinical Sciences, Auckland University of Technology, Auckland 0627, New Zealand; peter.mcnair@aut.ac.nz (P.M.); david.rice@aut.ac.nz (D.A.R.); 2 Department of Anaesthesiology and Perioperative Medicine, Waitemata District Health Board, Auckland 0620, New Zealand; Jill.Collier@waitematadhb.govt.nz

**Keywords:** chronic pain, musculoskeletal pain, osteoarthritis

## Abstract

Symptomatic hand osteoarthritis (OA) is a severely debilitating condition. Neuropathic pain (NP) has been shown to be a factor affecting pain severity, hand function, psychological wellbeing, body schema, and the number of pain medications in people with OA of other joints. The aim of this study was to assess the prevalence of NP in symptomatic hand OA and assess its association with pain, hand function, measures of psychological wellbeing, sleep, body schema disturbances, and number of pain medications. Participants with symptomatic hand OA diagnosed through the American College of Rheumatology criteria, were recruited and completed a series of online questionnaires. These included the Douleur Neuropathique 4 interview (DN4-interview), Short Form Brief Pain Inventory (SF-BPI), Neglect-like Symptoms questionnaire, Functional Index of Hand Osteoarthritis (FIHOA), Centre for Epidemiologic Studies Depression Scale (CES-D), Pain Catastrophising Scale (PCS), and the Pittsburgh Sleep Quality Index (PSQI). Logistic regression with age, body mass index, and sex as covariates were utilised to assess differences between participants with and without NP as identified through the DN4-interview. Correlation analysis assessed the relationship between pain intensity, body schema alterations, and number of pain medications. A total of 121 participants were included in the present study. Forty-two percent of participants presented with NP. Participants with NP reported higher levels of worst pain (OR: 10.2 95% CI: 2.2 to 48.5; *p* = 0.007). Worst pain intensity correlated with the number of pain medications (rho = 0.2; *p* = 0.04), and neglect-like symptoms (rho = 0.4; *p* < 0.0001). No difference between phenotypes was shown for catastrophising, function, depression, neglect-like symptoms, pain interference, or sleep. A large proportion of people with symptomatic hand OA present with NP. This phenotype is characterised by greater levels of pain intensity. Pain intensity is associated with number of pain relief medications and body schema alteration. Psychological factors, hand function, and sleep do not appear to be affected by the presence of NP.

## 1. Introduction

Symptomatic hand osteoarthritis (OA) affects 20% of people over the age of 70 [1]. Despite its large clinical burden, hand OA has been called “the forgotten disease”, receiving far less scientific attention than OA at other joints such as the knee and hip [2]. People with hand OA frequently seek medical care due to ongoing pain and disability [3]. It is therefore important to improve our ability to identify and classify different types of pain in order to develop more effective treatment plans [4].

Traditionally, OA has been described as a peripheral joint disease, with a strong nociceptive drive related to pain experience. Recent evidence has however suggested that features of neuropathic pain (e.g., burning pain, electric shocks) may also be present in subgroups of people with OA [5]. For example, in people with knee and hip OA [6], NP rates range between 20% to 70% of subjects [7,8]. Currently, it is unknown whether NP is common in people with hand OA. The identification of this pain phenotype in hand OA may be important as NP has been shown to be associated with greater levels of pain, worse function, greater pain catastrophising thoughts, higher levels of depression, and greater sleep disturbances in OA of other joints [7,9]. Considering that these characteristics are poor prognostic factors for musculoskeletal conditions [10], it is clear that identifying NP in clinical practice would be useful for clinicians. In addition, previous evidence has shown limited effectiveness of traditional analgesics in NP [6], suggesting that the first line pharmacological interventions consisting of non-steroidal anti-inflammatories (NSAIDs) and paracetamol may have very limited effectiveness in this subgroup of patients.

NP is not only associated with higher levels of pain but also brain related sensorimotor dysfunction [11]. Previous research has shown that body schema alterations are present in a large proportion of people with symptomatic hand OA [12] with 50% of participants presenting with neglect-like symptoms. It is possible that a large proportion of these people may also present with NP. Anecdotal evidence of a potential association between NP and neglect-like symptoms is provided by Oliver Sacks in their 1984 “A leg to stand on” book [13]. In their descriptions of recovery following a patellar tendon and femoral nerve injury, involuntary movements, and foreignness feelings of the affected limb were associated with a NP presentation. Experimental evidence of body schema alterations associated with deafferentation through anaesthesia have been reported by Türker et al. [14], and provide additional support to the hypothesis that NP may be associated with sensorimotor dysfunction in people with NP.

It was therefore the aim of this study to assess the prevalence of NP in people with hand OA and to determine whether pain intensity, pain interference, number of pain medications, hand function, neglect-like symptoms, psychological distress, and sleep quality were differentially associated with the presence of NP. In addition, we assessed the relationship between pain intensity, neglect-like symptoms, and the number of analgesic medications utilised. Finally, we were interested in the pain descriptors (DN4 descriptors) most commonly utilised by people with hand OA presenting with NP. Our main hypotheses were that: (1) participants with NP would present with higher pain intensity and interference compared to the non-NP group; (2) participants with NP would report taking a higher number of pain medications compared to the non-NP group; (3) participants with NP would present with lower hand function compared to the non-NP group; (4) participants with NP would present with higher neglect-like symptoms compared to the non-NP group; (5) participants with NP would present with higher psychological distress and sleep impairments compared to the non-NP group; (6) there would be a positive relationship between pain intensity and neglect-like symptom; (7) there would be a positive relationship between pain intensity and number of pain medications.

## 2. Materials & Methods

### 2.1. Study Design and Participants

This was a cross sectional study assessing the presence of NP in participants with hand OA. The inclusion and exclusion criteria are provided in Table 1. Our study was approved by the Auckland University of Technology Ethics Committees (AUTEC) and by the Health and Disability Ethics Committees (HDECs reference number: 16/CEN/191). All participants provided written informed consent. Participants were recruited by advertising on social media platforms, Arthritis New Zealand newsletters, and direct email to U3A (University of the Third Age, an older adult educational group) members. Initial screening and contact information was gathered by completion of a series of simple questions on the RedCap electronic data capture tools hosted at Auckland University of Technology [15,16]. These questions were opened by electronic link directly from the e-advert and enquired about hand pain, previous surgery, previous joint injections and the specific exclusion criteria. Potential participants who passed the initial screening were telephoned to be formally screened by the researcher (JC). Once consent was obtained, subjects were then automatically directed to the full series of online questionnaires.

### 2.2. Sample Size Calculations

An a-priori power calculation was performed to determine the sample size required to identify an association between the presence of high levels of pain (dichotomous independent variable: high vs. low) and NP (dichotomous dependent variable: yes vs. no) through binary logistic regression. Using G*power 3.1.9.7 software, the alpha level was set to 0.05, power to 0.80. The probability of participants presenting with high levels of pain and NP was set to 0.6 whilst the probability of high levels of pain with non-NP was set to 0.3 [5]. The amount of variability in pain levels explained by other covariates (R^2^) was set to 0.02. The proportion of participants expected to present with high levels of pain was set to 0.4 (40% of sample) as we predicted the number of participants with NP to be equal to or greater than 30% [8]. The hypothesis was set to two-tails. With these parameters, the sample size calculated was 93 participants.

### 2.3. Neuropathic Pain

NP was assessed through the DN4-interview. This questionnaire presents with seven items describing pain associated with neuropathic features. The maximum and minimum score for the tool is 7 and 0 respectively. This questionnaire has been previously validated against a pain specialist assessment in participants with painful diabetic polyneuropathy [17]. A score of 3 or greater, suggests the presence of neuropathic symptoms with a specificity and sensitivity of 84% [17]. The questionnaire has also shown good test and retest reliability with ICCs of 0.94 and 0.96 in non-NP and NP presentations [18].

### 2.4. Pain Intensity, Pain Interference, Neglect-like Symptoms, and Hand Function

Worst and average pain intensity was measured through questions one (worst pain in the last 24 h) and three (pain on average) of the Short Form Brief Pain Inventory (SF-BPI). These questions rated participants’ pain on an 11-point Numerical Rating Scale (NRS) with anchors of 0 = no pain and 10 = pain as bad as you can imagine. Pain interference (SF-BPI) was assessed by asking participants to rate the interference with general activity, mood, normal work, relations with other people, sleep and enjoyment of life on an 11-points NRS scale with anchors of 0 = does not interfere and 10 = completely interferes. The SF-BPI has been validated against the visual analogue scale for pain showing a moderate to large correlation (*r* > 0.6) and it presents with good test-retest reliability (*r* > 0.7) [19]. Neglect-like symptoms refer to difficulties in movement and perception of the affected limb. Neglect-like symptoms include feelings of disconnection with the affected arm, the need to focus attention towards the painful arm to make it move as intended, and the presence of involuntary movements. These symptoms are similar to those reported by people affected by parietal lobes lesions (e.g., stroke), which lead to hemineglect syndromes. However, in people with musculoskeletal pain these symptoms are not associated with objective neurological lesions, hence why the term “neglect-like symptoms” was introduced by Galer and Jensen et al. [20,21] to describe a similar set of symptoms. The presence of neglect-like symptoms was assessed through the Neurobehavioral Questionnaire [22]. This questionnaire asks participants to rate the frequency of each of the five items on a 1 to 6 Likert-scale with anchors of 1 = never and 6 = always (lowest score 5 and highest score 30). The Neurobehavioral Questionnaire has shown good reliability (Cronbach’α= 0.86) [22]. Hand function was measured with the Functional Index of Hand Osteoarthritis (FIHOA), which is a disease specific questionnaire [23]. The score on the FIHOA ranges from 0 to 30 with higher scores representing worse function. (FIHOA). The FIHOA has been validated against the Australian/Canadian Osteoarthritis Hand Index (AUSCAN) showing a strong correlation (*r* = 0.76), and excellent test re-test reliability (ICC: 0.94) [24].

### 2.5. Psychological Distress and Sleep

Psychological distress was assessed through the Centre for Epidemiologic Studies Depression Scale (CES-D) and the Pain Catastrophising Scale (PCS). The CES-D is a screening questionnaire for depressive disorders made of 20 items with scores ranging from 0 to 60 (higher scores associated with a greater level of depression). The CES-D has been shown to have moderate test-retest reliability (*r* > 0.45) [25], and it has been validated against a clinician’s diagnosis of depression [26]. The PCS investigates the emotions and the thoughts associated with the pain experience through 13 questions providing a score from 0 to 52 (higher scores associated with greater catastrophising thoughts and emotions). The PCS is a reliable questionnaire (Cronbach’α= 0.93) and has been validated against the Inventory of Negative Thoughts in Response to Pain (*r* = 0.56) [27]. To assess sleep, the Pittsburgh Sleep Quality Index (PSQI) was utilised. The PSQI is a 19 item with scores ranging from 0 to 21 (higher scores represent worst sleep). The PSQI has shown reasonable test-retest reliability (ICC > 0.8) and has been validated against other sleep questionnaires including the Insomnia Severity Index (*r* > 0.7) [28].

### 2.6. Statistical Analysis

Data were analysed using SPSS software version 26 (SPSS, Chicago, IL, USA), R version 4.0.4, and RStudio v 1.2.1335. Unadjusted logistic models were utilised to assess the association between pain, psychological distress, body schema, and sleep (independent variables) and the presence of NP. Adjusted logistic models (accounting for age, sex, and BMI as confounders [29]) were utilised to determine whether a correlation existed between the presence of NP and variables retained from the univariate analyses (*p* < 0.2). Missing data were removed for the univariate and multivariate analysis. The final model was then validated against multiple imputation datasets (five iterations). If the tested model performed equally on all the multiple imputation dataset, this was selected as the final model.

## 3. Results

### 3.1. Participants Characteristics

A total of 121 participants were eligible for inclusion in the study. The average age of the cohort was 69.6 years and 87% were female. Based on pain, participants appeared mildly to moderately affected by symptomatic hand OA (Table 2). Fifty-one participants (42%) reported ≥3 on the DN4 and were therefore classified as having neuropathic like pain.

### 3.2. Unadjusted Individual Effect of Pain Intensity, Pain Interference, Neglect-like Symptoms, and Hand Function on Neuropathic Pain

Participants who reported a greater number of medications, worst pain, pain interference, and neglect-like symptoms were more likely to present with NP (Table 3). Greater pain catastrophising appeared to increase the odds of NP despite it not being significant on an individual level (Table 3).

### 3.3. Effect of Worst Pain on Neuropathic Pain

When individual factors were included in a single model adjusted for confounders (age, sex, BMI), greater pain intensity (worst pain) was associated with greater odds of NP (Table 4). Additional adjusted models showed that the number of medications or neglect-like symptoms were significantly associated with the presence of NP albeit to a smaller extent than worst pain. A positive small and moderate correlation was identified between worst pain and number of pain medications (rho = 0.2; *p* = 0.04) and between worst pain and neglect-like symptoms (rho = 0.4; *p* < 0.0001).

### 3.4. Neuropathic vs. Non-Neuropathic Pain Characteristics

Forty-two percent of participants (*n* = 51) presented with NP symptoms. On average the NP group presented with four symptoms on the DN4, with the most common being “Tingling” (76.5%), “Burning pain” (67%), and “Pins and needles” (61%) (Table 5). Participants without NP, reported on average one symptom with the most frequent being “Burning pain” (*n* = 25/70; 36%) (Table 5).

## 4. Discussion

This is the first study assessing the prevalence of NP and its characteristics in people with symptomatic hand OA. Our results partially supported our hypothesis and showed that 42% of participants with hand OA presented with NP. In particular, the presence of NP was associated with higher levels of pain intensity (worst pain) but not pain interference. Hand function, psychological distress, or sleep were not affected by the presence of NP. Interestingly, the number of pain medications and neglect-like symptoms were higher in the NP group, but these did not identify the presence of NP as well as pain intensity did in our final models. Finally, as hypothesised, we identified a positive correlation between worst pain intensity, the number of pain medications, and neglect-like symptoms suggesting an association between symptoms, the quest for pain relief, and body schema alterations.

Our findings are in line with previous osteoarthritis research showing NP prevalence ranging between 20% to 67% in people affected by knee OA [8]. The high prevalence of NP is of importance due to its association with higher levels of pain and greater disease burden [30]. In the present study, “worst hand pain” was on average 1.7 points (NRS) greater in the NP group, which is close to the minimal clinically important difference for pain [31], compared to the non-NP group. It was also noted that pain severity was correlated with the amount of pain medications that participants were taking. It is however unclear whether taking a greater number of painkillers is beneficial, considering that previous research has found traditional analgesics to be less effective for NP [6].

Of interest was also the moderate correlation between pain intensity (worst pain) and neglect-like symptoms. The presence of neglect like symptoms has been previously reported in people with symptomatic hand OA [12] and its correlation with pain intensity has been demonstrated in subjects with chronic upper or lower limb pain [22,32]. The presence of neglect-like symptoms in chronic pain has been hypothesised to be due to an attentional bias away from the involved painful limb [33,34], body schema impairments secondary to cortical remodelling [35,36], or disuse [37]. An association between these mechanism and pain intensity is plausible and could therefore explain the association between neglect-like symptoms and pain severity.

Other variables such as depression, pain catastrophising, pain interference, hand function, or sleep were not affected by the presence of NP pain. Of note, however, sleep disturbances (PSQI) affected more than 70% of participants across the entire sample. This is a considerably higher proportion compared to the average percentage of older adults presenting with sleep disturbances which appears to vary between 52% [38] and 58% [39]. In addition, pain descriptors that characterised the NP group were “Tingling”, “Burning”, and “Pins and Needles”, which were reported by the 77%, 67%, and 61% of the group respectively. These descriptors are common in NP and have previously been reported by subjects with NP associated with knee OA [40].

Despite its large prevalence, the aetiology of NP pain in OA is not clear. Idiopathic small fibre neuropathy, which affects small C fibres as well as small myelinated Aδ fibres has been suggested as a potential cause of NP in osteoarthritis. In animal studies, models of osteoarthritis have shown the potential to reduce density of intra-epidermal nerve fibres, implying the presence of a NP component to osteoarthritis [41]. However, there is currently no evidence to support these findings in humans. Altered nociception has also been proposed as a mechanism explaining NP symptoms in rheumatological condition [6]. Pain arising under these circumstances may lead to an increase in excitability of nociceptors and activation of descending facilitatory pathways, which in turn can cause pain hypersensitivity and increase pain intensity [6]. We are however unable to confirm or disprove any of these causal mechanisms as it was not the purpose of this study to elucidate the origin of NP in hand OA. Further research is therefore required to shed light on this topic.

This study is not without its limitations. First, we were unable to objectively identify the presence of NP. A pain specialist assessment of subjective and objective findings is considered the gold standard for the diagnosis of NP. Such assessment was not performed and we believe that although this is a limitation, the use of a questionnaire is more representative of how clinicians would assess clients with symptomatic hand OA in clinical practice. In addition, due to the nature of the study, the correlations identified do not prove any causation and a third unknown variable could be driving the associations described. Thirdly, we did not classify the type of painkillers participants were taking, but only the total number. In addition, our sample largely consisted of participants with mild to moderate hand OA. It is possible that more severe forms of hand OA would lead to different results. Finally, our sample of symptomatic hand OA participants was heterogenous and we did not specifically focus on a subgroup of clients (e.g., basal thumb OA).

In summary, this study provides evidence that a large proportion of people with symptomatic hand OA present with NP. This phenotype is associated with greater levels of pain, which is associated with greater consumption of analgesics and body schema alterations. Future studies may wish to assess the effectiveness of conservative and pharmacological interventions on this phenotype of symptomatic hand OA.

## Figures and Tables

**Table 1:** Inclusion and exclusion criteria for participants’ recruitment.

Inclusion Criteria	Exclusion Criteria
Older than 18 years old Fulfils ACR criteria: Hand pain, aching, or stiffness and 3 or 4 of the following: - Hard tissue enlargement of 2 or more of 10 selected joints * - Hard tissue enlargement of 2 or more DIP joints - Fewer than 3 swollen MCP joints - Deformity of at least 1 of 10 selected joints * Radiographic evidence of hand OA Hand pain for at least three months on consecutive days in the last year Hand pain in the week before testing scored at three or higher on an 11-point verbal NRS	Past or present Hx of neurological disease Hx of infection in the last three months Surgery to the hands in the last one year or joint injection in the last three months Uncontrolled hypertension Active cancer Diabetes Radiculopathy Rheumatological conditions

Note. ACR, American College of Rheumatology; MCP, Metacarpophalangeal; Hx, History; NRS, numeric pain rating scale; *, second and third distal interphalangeal (DIP), the second and third proximal interphalangeal, and the first carpometacarpal joints of both hands.

**Table 2:** Participant characteristics. Presented as mean (SD), number (proportion) or median (range).

	NP Group (*n* = 51)	Non-NP Group (*n* = 70)	Combined Group (*n* = 121)
Age (years)	69.3 (11.7)	70 (8.5)	69.9 (10)
Females	45 (88%)	60 (86%)	105 (87%)
Right hand dominant	45 (88%)	61 (87%)	106 (88%)
Height (m)	1.63 (0.1)	1.65 (0.1)	1.64 (0.1)
Mass (kg)	69.6 (13.9)	70.3 (17.9)	70 (16)
BMI (kg/m^2^)	26.1 (5.3)	25.5 (5)	25.8 (5.1)
Right hand most painful	24 (47%)	29 (41%)	53 (44%)
Bilateral hand pain	14 (28%)	16 (23%)	30 (25%)
Number of other painful sites	3.4 (2.8)	2.4 (2)	2.8 (2.5)
Duration of pain (years)	9.1 (9.6)	8.9 (7)	9 (8.2)
Number of pain medications	1 (0 to 4)	1 (0 to 5)	1.18 (1.1)
DN4	3.9 (0.9)	1.1 (0.8)	2.3 (1.6)
Average hand pain (NRS)*	3.8 (1.7)	3.3 (1.7)	3.5 (1.7)
Worst hand pain (NRS)*	6.1 (2.2)	4.4 (2.2)	5.1 (2.4)
SF-BPI (Total)	2.8 (1.9)	1.9 (1.8)	2.3 (1.9)
Neglect-like symptoms	7.9 (3.2)	6.8 (2.7)	7.2 (2.9)
FIHOA (0 to 100)	13.9 (8)	14.9 (8.9)	14.5 (8.5)
CES-D	10.8 (9)	8.9 (8.7)	9.7 (8.9)
PCS	10.6 (10)	6.8 (6.2)	8.4 (8.3)
PSQI	7.3 (3.9)	7.2 (3.6)	7.2 (3.7)
Poor sleep (PSQI ≥ 5)	37/51 (73%)	49/70 (70%)	86/117 (71%)

Note. NP, Neuropathic pain; BMI, body mass index; DN4, Douleur Neuropathique in 4 questions; NRS, numerical pain rating scale (0–10, where 0 = “no pain” and 10 = “pain as bad as you can imagine”); *, in most painful hand; SF-BPI, Short Form Brief Pain; FIHOA, Functional Index of Hand Osteoarthritis; CES-D, Centre for Epidemiologic Studies Depression Scale; PCS, Pain Catastrophising Scale; PSQI, Pittsburgh Sleep Quality Index.

**Table 3:** Associations between demographics, pain, psychological distress, sleep and neuropathic pain in people with hand OA (sample of 105 participants—missing data were removed).

	Total Sample		OR	95% CI Lower	95% CI Upper	*p* Value
	*n*	%				
Age						
18–64 years old	25	23.8%	1			
65–70 years old	21	20.0%	0.29	0.08	1.03	
71–76 years old	33	31.4%	0.98	0.35	2.77	
77–89 years old	26	24.8%	0.58	0.19	1.76	0.15
Sex						
Male	14	13.3%	1			
Female	91	86.7%	1.41	0.44	4.54	0.56
BMI						
Normal	54	51.4%	1			
Overweight	27	25.7%	1.36	0.53	3.48	
Obese	24	22.9%	2.01	0.76	5.32	0.36
Number of other painful sites						
0	15	14.3%	1			
1–2	46	43.8%	1.07	0.31	3.66	
3	14	13.3%	2.00	0.45	8.96	
4 or more	30	28.6%	2.62	0.72	0.15	0.22
Duration of pain (years)						
0–2 years	26	24.8%	1			
3–5	28	26.7%	0.4	0.14	1.21	
6–11	30	28.6%	0.27	0.09	0.81	
12 and over	21	20.0%	0.47	0.15	1.51	0.12
Number of pain medications						
0	36	34.3%	1			
1	36	34.3%	4.2	1.54	11.46	
2	22	21.0%	1.4	0.43	4.53	
3 or more	11	10.5%	8	1.74	36.81	0.003
Average hand pain (NRS)						
0–1	13	12.4%	1			
2	24	22.9%	5.5	1.00	30.28	
3–4	43	41%	4.35	0.86	22.05	
5–10	25	23.8%	5.08	0.93	27.75	0.15
Worst hand pain						
0–2	17	16.2%	1			
3–4	27	25.7%	2.33	0.53	10.27	
5–6	30	28.6%	3.57	0.84	15.08	
7–10	31	29.5%	8.48	1.99	36.09	0.008
SF-BPI						
0	37	35.2%	1			
1–2	37	35.2%	2.95	1.1	7.93	
3 or over	31	29.5%	4.31	1.53	12.14	0.012
Neglect-like symptoms						
No	42	40%	1			
Yes	63	60%	2.75	1.2	6.32	0.015
FIHOA						
0–9	33	31.4%	1			
10–19	40	38.1%	1.2	0.48	3.02	
20 or over	32	30.5%	0.55	0.2	1.5	0.26
CES-D						
No	82	78.1%	1			
Yes	23	21.9%	1.29	0.51	3.28	0.59
PCST						
0–2	29	27.6%	1			
3–5	24	22.9%	2.22	0.71	6.97	
6 or over	52	49.5%	2.62	0.99	6.99	0.13
PSQI						
0–3	16	15.2%	1			
4–6	32	30.5%	1.3	0.38	4.43	
7–9	27	25.7%	1.55	0.44	5.47	
10 or over	30	28.6%	1.11	0.32	3.87	0.90

Note. OR, odds ratio; CI, confidence interval; NP, Neuropathic pain; BMI, body mass index; DN4, Douleur Neuropathique in 4 questions; NRS, numerical pain rating scale (0–10, where 0 = “no pain” and 10 = “pain as bad as you can imagine”); SF-BPI, Short Form Brief Pain; FIHOA, Functional Index of Hand Osteoarthritis; CES-D, Centre for Epidemiologic Studies Depression Scale; PCS, Pain Catastrophising Scale; PSQI, Pittsburgh Sleep Quality Index.

**Table 4:** Multiple logistic regression assessing the effect of pain on the risk of neuropathic pain in people with hand OA.

	Total Sample		OR	95% CI Lower	95% CI Upper	*p* Value
	*n*	%				
Age						
18–64 years old	25	23.8%	1			
65–70 years old	21	20.0%	0.35	0.09	1.41	
71–76 years old	33	31.4%	1.53	0.45	5.21	
77–89 years old	26	24.8%	0.57	0.17	1.9	0.11
Sex						
Male	14	13.3%	1			
Female	91	86.7%	1.41	0.39	5.04	0.60
BMI						
Normal	54	51.4%	1			
Overweight	27	25.7%	1.52	0.50	4.62	
Obese	24	22.9%	1.92	0.66	5.57	0.44
Worst hand pain						
0–2	17	16.2%	1			
3–4	27	25.7%	2.25	0.46	11.03	
5–6	30	28.6%	4.3	0.88	20.92	
7–10	31	29.5%	10.23	2.16	48.51	0.007

Note. OR = odds ratio; CI = confidence interval.

**Table 5:** Neuropathic symptoms frequency in the most painful hand of participants with and without neuropathic pain.

	NP Group (*n* = 51)	Non-NP Group (*n* = 70)	Combined Group (*n* = 121)
Burning pain	34 (67%)	25 (36%)	59 (49%)
Painful cold	19 (37%)	15 (21%)	34 (28%)
Electric shocks	30 (59%)	11 (16%)	41 (34%)
Tingling	39 (76.5%)	7 (10%)	46 (38%)
Pins and needles	31 (61%)	3 (4%)	34 (28%)
Numbness	30 (59%)	13 (19%)	43 (36%)
Itching	17 (33%)	3 (4%)	20 (17%)

Note. NP, neuropathic pain.

## Data Availability

Requests for data collected in the HOPE study (such as deidentified participant data) can be made to the corresponding author following publication, and requests will be considered on an individual basis.
